# Salivary Metabolomics in Patients with Long COVID-19 Infection

**DOI:** 10.3390/metabo14110598

**Published:** 2024-11-07

**Authors:** Luiz Machado, Robson Prudente, Estefânia Franco, Mariana Gatto, Gustavo Mota, Luana Pagan, Luís Brizola, Maércio dos Santos, Thulio Cunha, Robinson Sabino-Silva, Luiz Goulart, Mario Martins, Paula Santos, Larissa Maia, André Albuquerque, Eloara Ferreira, Bruno Baldi, Marina Okoshi, Suzana Tanni

**Affiliations:** 1 Botucatu Medical School, São Paulo State University, Botucatu 18618-970, Brazil; robson.prudente@unesp.br (R.P.); estefania.franco@unesp.br (E.F.); mariana.gatto@unesp.br (M.G.); gustavo.mota@unesp.br (G.M.); luanapagan@alunos.fmb.unesp.br (L.P.); lfpb@uninove.edu.br (L.B.); maercio.santos@unesp.br (M.d.S.); mpoliti@fmb.unesp.br (M.O.); suzana.tanni@unesp.br (S.T.); 2 Faculty of Medicine of the Federal, University of Uberlandia, Uberlândia 38408-100, Brazil; thulio.cunha@ufu.br (T.C.); robinsonsabino@ufu.br (R.S.-S.); mario.martins@ufu.br (M.M.); paulasantos.bio@gmail.com (P.S.); larissa.maia@uemg.br (L.M.); 3 Department of Pneumology, University of São Paulo, São Paulo 05403-000, Brazil; drean.albuquerque@gmail.com (A.A.); bruno.baldi@hc.fm.usp.br (B.B.); 4 Department of the Federal, University of São Paulo, São Paulo 01246-903, Brazil; eloara.ferreira@unifesp.br

**Keywords:** COVID-19, post-COVID-19, metabolomics, functional capacity

## Abstract

**Background:** Long COVID-19 has been characterized by the presence of symptoms lasting longer than 4 weeks after the acute infection. The pathophysiology of clinical manifestations still lacks knowledge. **Objective:** The objective of this paper was to evaluate metabolite abundance in the saliva of long COVID patients 60 days after hospital discharge. **Methods:** A convenience sample was composed of 30 post-discharge patients with long COVID and seven non-COVID-19 controls. All COVID-19 patients were evaluated by demographic characteristics, spirometry, 6 min walk test (6mWT), Saint George Respiratory Questionnaire (SGRQ), and body composition. Metabolomics was performed on saliva. **Results:** The long COVID-19 patients were 60.4 ± 14.3 years-old, and 66% male. Their lean body mass was 30.7 ± 7.3 kg and fat mass, 34.4 ± 13.7 kg. Spirometry evaluation showed forced vital capacity (FVC) of 3.84 ± 0.97 L with 96.0 ± 14.0% of the predicted value, and forced expiratory volume in the first second (FEV_1_) of 3.11 ± 0.83 L with 98.0 ± 16.0 of the predicted value. The long COVID-19 patients had reduced maximal inspiratory (90.1 ± 31.6 cmH_2_O) and maximal expiratory (97.3 ± 31.0 cmH_2_O) pressures. SGRQ showed domain symptoms of 32.3 ± 15.2, domain activities of 41.9 ± 25.6, and domain impact 13.7 ± 11.4, with a mean of 24.3 ± 14.9%. Physical capacity measured by distance covered in the 6mWT was 418.2 ± 130 m with a 73.3% (22.3–98.1) predictive value. The control group consisted of 44.1 ± 10.7-year-old men with a body mass index of 26.5 ± 1.66 Kg/m^2^. Metabolomics revealed 19 differentially expressed metabolites; expression was lower in 16 metabolites, and 2 metabolites were absent in the COVID-19 patients compared to controls. Calenduloside G methyl ester (*p* = 0.03), Gly Pro Lys (*p* = 0.0001), and creatine (*p* = 0.0001) expressions were lower in patients than controls. **Conclusions:** Long COVID-19 patients present less abundance of calenduloside G methyl ester, Gly Pro Lys, and creatine in saliva than healthy controls. Lower creatine abundance may be related to reduced physical capacity and fatigue

## 1. Introduction

The World Health Organization (WHO) [1] officially recognized COVID-19 as a pandemic on 11 March 2020. As of 31st March 2024, more than 774 million cases have been globally notified with more than seven million deaths. Common symptoms of COVID-19 are fever, cough, chest pain, dyspnea, asthenia, and myalgia [2,3]. Risk factors for disease severity and mortality include advanced age, male gender, obesity, high blood pressure, and diabetes [2,4,5,6]. Epidemiological data from Brazil showed a 38% mortality rate in hospitalized patients, which reached 80% in patients under mechanical ventilation [7].

The pandemic led to urgent studies on COVID-19 etiology and prognosis. However, the mechanisms involved in different long-term COVID-19 sequelae have received little attention [8]. Changes in cellular immune response and cytokine storm play a crucial role in the pathogenesis, clinical manifestation, and prognosis of the disease [9]. Several terms have been used to describe persistent post-COVID-19 symptoms. More recently, long COVID was defined as the presence of symptoms lasting more than 4 weeks after acute infection, and post-COVID-19 condition when symptoms persist over 3 months after infection, last more than 2 months, and are not explained by another disease [10,11]. Manifestations of long COVID-19 can involve skeletal muscles and respiratory, cardiovascular, neurological, and gastrointestinal systems.

Long COVID symptoms are frequent. Approximately 70% of individuals can present dyspnea and 60% fatigue [12]. Carfi et al. (2020) [13] observed that 70% of individuals with one or more symptoms were physically fit before the disease but became sedentary after COVID-19. Symptoms such as fatigue, dyspnea, chest pain, muscle weakness, sleeping disturbance, anxiety, and depression have been described and can impact general health status and public health system [14,15].

Long COVID syndrome can reduce physical capacity. A study evaluating 1,250 long COVID patients showed that 60 days after hospital discharge, 6.7% of patients had died and 15.1% required hospital readmission for long COVID complications [16]. Another study with 143 individuals observed that 87% had one or more symptoms 60 days after hospital discharge, with 44% presenting reduced quality of life, 53% fatigue, and 43% dyspnea [13].

To assess potential mechanisms involved in long COVID-19, different biological analyses have been employed [17,18]. Salivary biomarkers can be evaluated using RNA sequencing, liquid biopsy, fluorescent biosensors, photometric and electrochemical methods, and electric-field-induced release measurement [17,18,19]. Saliva is produced and secreted by the parotid, submandibular, and sublingual glands, which are richly surrounded by circulating blood. This facilitates molecule exchange between blood and salivary acini. Virus present in the lower respiratory tract, nasopharynx, and salivary glands may be found in saliva; therefore, it can be used to diagnose disease [18,20,21,22,23]. The presence of angiotensin-converting enzyme (ACE) receptors in salivary glands indicates their potential role in SARS-CoV-2 replication [24,25]. 

Several studies have applied omics analysis to identify biomarkers in plasma [25,26,27]. Researchers have suggested that metabolite profile in serum and plasma is useful in stratifying mild to severe COVID-19 cases [28,29]. Metabolomics offers insights into the biochemical pathways impacted by SARS-CoV-2, its treatment, and the metabolites associated with long COVID-19 symptoms. By examining biomarkers of cellular metabolic processes, metabolomics sheds light on the disease pathophysiology. Therefore, identifying changed metabolites abundance may lead to new treatments for long COVID-19. The aim of this study was to analyze saliva metabolites in patients with long COVID-19 60 days after hospital discharge. These results were compared to healthy, never infected with SARS-CoV-2, non-smoker individuals.

## 2. Materials and Methods

This is a cross-sectional observational study, carried out in accordance with the guidelines of the Declaration of Helsinki, and approved by the Ethics Committee of the Botucatu Medical School, UNESP, Brazil (Proc. n. 312588/2020), on 22 July 2020. All COVID-19 patients discharged from Botucatu Medical School Clinical Hospital were referred to the long-term COVID-19 follow-up clinic.

Eligibility criteria for long COVID patients were age over 18 years old and the presence of at least one of the following symptoms: fever, cough, myalgia, and dyspnea 60 days after hospital discharge. During the hospitalization, patients should have had a positive RT-PCR test for COVID-19 and required oxygen supplementation via nasal catheter, invasive or non-invasive mechanical ventilation, or non-rebreathing mask. Patients diagnosed with another associated virus were excluded from the study. 

At the first medical evaluation 60 days after hospital discharge, patients were invited to participate in the study and signed the Informed Consent Form. The following information was assessed: demographic data, hospital history of disease severity, body composition, quality of life, and dyspnea severity. Pulmonary function, respiratory strength, peripheral muscle strength, physical exercise capacity through 6 min walk test (6mWT), arterial blood gas, D-dimer, and white blood count were also evaluated. Saliva was collected from both long COVID-19 patients and healthy controls.

### 2.1. Electrical Bioimpedance

Body composition was assessed by multifrequency electrical bioimpedance using the BC-558 Ironman Tanita or Inbody 720 analyzers. The following variables were measured: body mass (Kg), body mass index (BMI), percentage of body fat tissue, total muscle mass (Kg), and appendicular muscle mass (Kg), which is the sum of the each limb muscle mass.

### 2.2. Quality of Life and Dyspnea Severity Questionnaires

Health status was analyzed using the validated Brazilian language and culture version of the Saint George’s Respiratory Questionnaire (SGRQ), composed of three domains: symptoms related to respiratory symptoms; impact of symptoms on life activities and well-being; and changes in physical activity [30]. Dyspnea was assessed using the Modified Medical Research Council (MMRC) scale [31] and the Baseline Dyspnea Index (BDI) [32]. Both questionnaires were the translated Portuguese language and culture version. 

### 2.3. Arterial Blood Gases

Arterial blood was collected from the radial artery and blood gases analyzed using a Stat Profile 5 Plus gas analyzer (Nova Biomedical, Waltham, MA, USA).

### 2.4. Lung Function

Spirometry was performed on a portable computerized pulmonary function system (Ferraris Koko, Louisville, CO, USA) according to American Thoracic Society criteria [33]. Measurements were obtained 20 min after using a fenoterol bronchodilator (400 mcg). Bronchodilator inhalation was performed with the patient sitting, inhaling four puffs of fenoterol up to total lung capacity (TLC) four times. The patient should maintain TLC for 10 s.

### 2.5. Six-Minute Walk Test

The 6mWT was performed according to American Thoracic Society guidelines [34]. The tests were performed without oxygen supplementation. Desaturation was characterized by a drop in peripheral oxygen saturation (SpO_2_) to less than 88% for more than 1 min. Dyspnea and lower limb fatigue were assessed by the modified BORG scale after 6mWT [35].

### 2.6. Severity of the Acute Infection

Acute COVID-19 severity was assessed by the following: 4C Mortality Score, which was validated in the ISARIC World Health Organization Clinical Characterization Protocol UK study [36]; sequential organ failure assessment (SOFA) [37]; simplified acute physiology score version 3 (SAPS III) [38]; and acute physiology and chronic health evaluation (APACHE II) [39].

### 2.7. Metabolomics Analysis

Saliva samples were obtained from 30 patients and 7 healthy donors. Saliva was collected using a commercially available collection system (Salivette^®^, Sarstedt, Numbrecht, Germany). After rinsing the mouth with water, 2 mL of saliva was obtained under a protease inhibitor solution. Samples were centrifuged (Thermo Fisher^®^, Thermo Fisher Scientific, Bedford, MA, USA) at 10,000× *g* for 10 min at 4 °C and supernatant stored at −80 °C.

Saliva metabolomics was performed at the Federal University of Uberlândia, Brazil. For metabolite extraction, 100 µL of sample was added to 1000 µL of spectroscopic grade methanol and the mixture incubated for 4 h at −80 °C. It was then centrifuged for 15 min at 13,000× *g* and the supernatant transferred to another microtube, which was concentrated in a vacuum concentrator for 30 min and lyophilized. The material was stored at −80 °C until analysis. For mass spectrometry (MS) analyses, samples were suspended in 500 µL of spectroscopic grade methanol and filtered through a 0.22 µm filter.

The high-performance liquid chromatography/mass spectrometer (HPLC/MS) (Agilent^®^, Agilent Technologies, Santa Clara, CA, USA) analyses were performed using an Agilent Infinity 1260 liquid chromatograph (Agilent^®^, Agilent Technologies, Santa Clara, CA, USA) coupled to a type Q-TOF model 6520 B high-resolution mass spectrometer with an electrospray ionizaton (IES) source (Agilent^®^, Agilent Technologies, Santa Clara, CA, USA). The chromatographic parameters were as follows: Agilent Poroshell column model Zorbax C18, 2.1 mm internal diameter, 5 cm length, 2.7 μm particles (Agilent®, Agilent Technologies, Santa Clara, CA, USA); mobile phase: water acidified with formic acid (Sigma-Aldrich®, Merck KGaA, Darmstadt, Alemanha) (0.1% *v*/*v*) (A) and methanol (B) (J.T.Baker®, Avantor Performance Materials, Xalostoc, Mexico), with the gradient 2% B (0 min), 98% B (0–20 min); 98% B (6–12 min) with injection of 2 µL. Ionization parameters were as follows: 25 psi nebulizer pressure and 8 L.min^−1^ drying gas 220 °C; 4.5 KVa capillary energy. The samples were acquired in positive mode with data storage in centroid and profile. Constituent identification was carried out considering high-resolution mass, exact mass error less than 10 ppm compared to the Metlin Library, and the literature.

### 2.8. Statistical Analysis

Sample size was not calculated due to the lack of data on metabolomics in this population. A convenience sample with at least 30 long COVID-19 patients and 7 healthy subjects never infected with COVID-19 was considered.

For the analysis of metabolomics data, the acquired mass spectra were extracted using the MassHunter Qualitative v. 7.0 0 (Agilent^®^, Agilent Technologies, Santa Clara, CA, USA) program and converted. CEF extension, and the files were submitted to the MassHunter Mass Profiler Professional v. B.13.1.1(Agilent^®^, Agilent Technologies, Santa Clara, CA, USA) program. Mann–Whitney test and Bonferroni post hoc test with *p* < 0.05 and fold change greater than or equal to 2.0 were used to identify differences in the abundance of metabolites between the groups. With these compounds, a Partial Least Squares discriminant analysis (PLS-DA regression) was conducted.

## 3. Results

Thirty patients were included in the long COVID group and 7 in the control group. During hospitalization, 4 (13%) of the long COVID patients required invasive mechanical ventilation and 3 (10%) renal replacement therapy (Table 1). Long COVID patients were predominantly male (66%), and aged 60.4 ± 14.3 years old. Most healthy subjects were male (57%), 44 years old, and with a body mass index of 26.5 ± 1.66 Kg/m^2^.

Data obtained at the first post-hospital discharge visit are shown in Table 2. At bioimpedance, skeletal muscle mass and fat-free muscle mass had a mean of 30.7 ± 7.3 and 34.4 ± 13.7 kg, respectively. Patients reached 85.8% of the predicted maximal inspiratory pressure (MIP) with a mean of 97.3 ± 31.1 cm H_2_Oand 87% of predicted maximal expiratory pressure (MEP) with a mean of 87.6 ± 17.5 cm H_2_O. Patients presented forced vital capacity (FVC) of 3.84 ± 0.97 L (96 ± 14.3% of predicted value), forced expiratory volume in the first second (FEV_1_) of 3.11 ± 0.83 L (98% ± 15.9% of predicted value), and FVC/FEV_1_ ratio of 0.8 ± 0.6%. Dyspnea frequency and severity were evaluated by the MMRC scale and BDI. MMRC scale grade frequencies were 43% of patients Grade 0, 36% Grade 1, 10% Grade 2, and 10% Grade 3. Other results are shown in Table 2.

Quality of life and functional capacity, evaluated using the SGRQ, showed the following values: domain symptoms 32.3 ± 15.2, domain activities 41.9 ± 25.6, and domain impact 13.7 ± 11.4, with a mean of 24.3 ± 14.9%. Results from laboratory tests and arterial gases are presented in Table 3.

**Figure 1:**
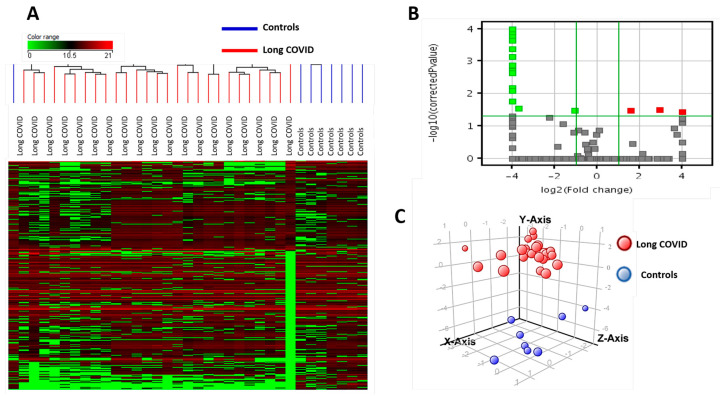
Salivary metabolomic analysis. (**A**) Hierarchical clustering heatmap showing metabolites differentially abundant between long COVID patients and controls. Log2 represents sample data included in the software, which were normalized in base 2 and presented as columns; metabolites are shown as lines. Separation between long COVID and control individuals is represented by the columns, red for controls and blue for long COVID patients. (**B**) is a volcano plot that shows the discrimination 19 metabolites with significant difference between long COVID and Controls subjects; (**C**) Supervised partial least squares discriminant analysis (PLS-DA) shows the discrimination between long COVID (red circles) and control (blue circles) subjects.

In metabolomics, a total of 5288 compounds and 338 metabolites were identified in the salivary samples. We used a frequency filter of 75% (only compounds found in 75% of the biological samples within at least one group were considered) to assure a quality control of the data (Figure 1A). The volcano plot shows the discrimination 19 metabolites with significant difference between long COVID and controls subjects (Figure 1B). After applying Mann–Whitney test and Bonferroni post hoc test with *p* < 0.05 and fold change greater than or equal to 2.0, we observed that the abundance of 19 metabolites differed between long COVID patients and controls. With these, a Partial Least Squares discriminant analysis (PLS-DA regression) was conducted (Figure 1C).

**Figure 2:**
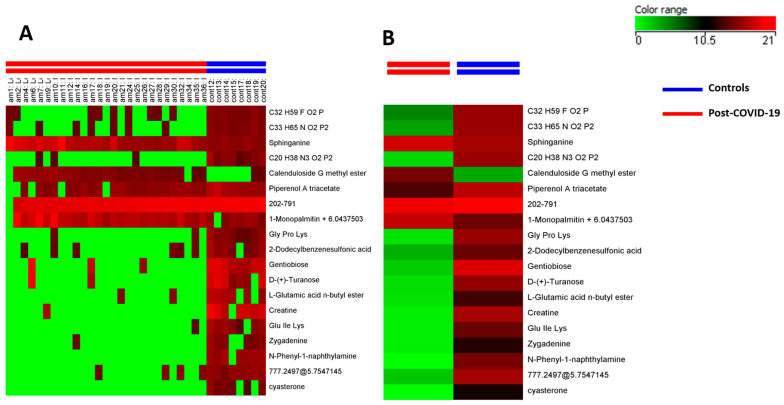
Metabolites with significant difference between long COVID patients and controls. (**A**) Heatmap showing the metabolite abundance. Lines represent the 19 metabolites which had a different abundance between groups; columns represent the long-COVID and control groups. Color intensity expresses metabolite abundance: green color represents metabolites more abundant and red color metabolites less abundant. (**B**) Heatmap with media showing the abundance of the 19 metabolites that differed between groups. The red column represents data from the long COVID, and the blue column represents data from the control group.

Figure 2 shows metabolites in long COVID patients and controls as a heatmap (with average and individual heatmaps) to illustrate the metabolite abundance that differed between the groups. Sphinganine, piperenol A triacetate, and 1-monopalmitin did not differ between groups. The metabolites Gly Pro Lys, 2-dodecyl benzene sulfonic acid, gentiobiose, D-(+)-turanose, L-glutamic acid n-butyl ester, creatine, Glu IIe Lys, zygadenine, N-phenyl-1-naphthylamine and cyasterone were significantly less abundant in long COVID-19 subjects than in controls, whereas calenduloside G methyl was more abundant in the long COVID patients.

## 4. Discussion

This study analyzed saliva metabolites in patients with long COVID-19 60 days after hospital discharge and compared the results to those from healthy, never-SARS-CoV-2-infected individuals. The main results showed that long COVID-19 patients had lower levels of metabolites calenduloside G methyl ester, Gly Pro Lys, and creatine in saliva than healthy controls.

We detected 338 metabolites, of which 19 showed a significantly different abundance between groups. Two metabolites, N-phenyl-1-naphytlamine and cyasterone, were absent in the long COVID group. Cyasterone has an antitumor effect in skin cancer and cytotoxic properties in several cell lines; it also stimulates osteogenesis and may accelerate fracture healing [40]. Calenduloside E 6′-methyl ester was only found in the long COVID group. It belongs to the family of oleanane-type triterpenoids. This metabolite has anticancer and anti-inflammatory properties, and prevents oxidative stress and hypertension [41,42,43]. Lee et al. [44] observed that calenduloside E 6′-methyl ester induces apoptosis and inhibits tumor growth in CT-26 mouse colon carcinoma in both in vitro and in vivo studies.

COVID-19 is accompanied by cardiovascular changes such as myocarditis, acute myocardial infarction, and venous and arterial thromboembolism. Gly Pro Lys was less abundant in long COVID patients than controls. This metabolite is a peptide involved in the inhibition of the angiotensin-converting enzyme (ACE), which might explain some cardiovascular changes in long COVID-19 patients [24].

Gentiobiose is a disaccharide composed of one glucose molecule linked to another via a beta (1→6) linkage. This bond distinguishes gentiobiose from maltose, which has an alpha (1→4) linkage. Commonly found in plants, gentiobiose is a component of polysaccharides such as acellulose and hemicellulose. It is also a major component of gentio-oligosaccharides (GnOS), a specific category of oligosaccharides. These have been recognized for their resistance to digestion by both common oral bacteria and intestinal enzymes and classified as difficult-to-digest sugars [45,46]. The molecular structure of GnOS, which includes hard-to-break glycosidic bonds, enhances their probiotic effects in the intestine, promoting a favorable bacteria environment.

D-(+)-Turanose, a sucrose isomer and disaccharide carbohydrate composed of glucose and fructose molecules, is commonly found in honey [47]. Unlike other simple sugars, turanose is hydrolyzed more slowly in the intestine, releasing glucose and fructose gradually. This process contributes to its lower glycemic index than other sugars, offering a healthy alternative for maintaining physiological blood glucose levels [48,49,50].

Creatine (Cr) is an important metabolite that was differentially abundant between the groups. Creatine, derived from amino acids and synthesized in the liver, pancreas, and kidneys from glycine, arginine, and methionine, was significantly lower in the long COVID patients. Creatine biosynthesis is initiated by the formation of guanidinoacetate (GAA) from arginine and glycine catalyzed by glycine aminotransferase. Subsequently, guanidinoacetate methyltransferase methylates GAA to produce creatine. Glycine has a role in both the Krebs cycle and metabolic processes. Although classified as a non-essential amino acid as the body can synthesize it under healthy conditions, glycine becomes conditionally essential during specific scenarios such as inflammation and intense physical activity, where its endogenous production is reduced, and dietary supplementation is necessary. Creatine resides in muscle cells and plays an important role in high-energy phosphate storage and transport, crucial for cell energy regulation [51]. When transformed into creatine phosphate, it acts as a phosphate donor in the conversion of adenosine diphosphate (ADP) to adenosine triphosphate (ATP), supplying the energy for muscle contraction. This conversion is facilitated by the creatine kinase/phosphocreatine (CK/PCr) transport system, which provides immediate ATP replenishment through high-energy phosphate compounds [51]. The ability of creatine to store energy in the form of PCr is indispensable for ATP synthesis, serving as an indirect energy reserve for cells and tissues. ATP synthesis ensures that oxidation occurs concurrently with new ATP generation, allowing PCr to release stored energy as needed, particularly under conditions of oxygen and glucose deficit [51,52].

PCr is also essential for maintaining neuron metabolism. In contrast to muscle cells, neurons lack the capacity to store glycogen, making the CK/PCr system essential for energy buffering and regulation. When this system is absent, neurons face detrimental changes in their intracellular environment. Decreased creatine levels may activate cellular proteins and enzymes and lead to adverse effects, such as intracellular acidification and ATPase inactivation, compromising cell energy balance and cell function [51]. Creatine also acts on the immune system, particularly in T cell activation, playing a vital role in the immune response against infections [51].

The lower creatine abundance in long-COVID-patient saliva suggests a possible relationship between reduced physical capacity and fatigue and lower creatine levels. However, this still needs further exploration to understand the mechanisms related to the changed metabolic pathway and symptomatic manifestation. Saito et al. [53] identified 18 altered metabolic pathways in plasma of long COVID patients. The changes persisted 12 months after the acute phase of the disease and included significant reduction in the abundance of aspartate, uracil, serine, sarcosine, arginine, dehydroalanine, thymine, and porphobilinogen. In patients with long COVID, as in our study, no oxygen deficit was observed in arterial blood gas analysis or in the exercise test (6 min walk test, 6mWT) [54,55,56].

To the best of our knowledge, this is the first study evaluating saliva metabolomics in patients with long COVID-19. Our data allow us to hypothesize that dietary supplementation focused on improving energy metabolism is beneficial for long COVID patients. Currently, the only recommendation for recovery and symptoms reduction for long COVID patients is physical activity and cardiopulmonary/motor rehabilitation. Even with non-pharmacological treatment, the symptomatologic recovery period is still prolonged [57]. In a study carried out by Benedetto et al. [58], chronic obstructive pulmonary disease (COPD) patients improved 6mWT, dyspnea index, and daily living activities score after creatine supplementation and rehabilitation.

As metabolic pathways can be changed by diet, a limitation of this study is that the dietary history of our patients and controls could not be obtained at the time of evaluation. The results of this study were approached descriptively, independent of age, BMI, and body composition. Another limitation refers to the fact that we have not included a cohort of patients that were infected but have fully recovered from COVID-19. As we had not previously collected saliva from all hospitalized COVID patients, it was not possible to include this cohort in our study.

Finally, as an exploratory study, no metabolic pathways could be established in this work. Therefore, additional studies are needed to clarify metabolic pathways and to elucidate the pathophysiology involved in the symptom persistence in long COVID-19 patients.

## 5. Conclusions

Abundance of nineteen salivary metabolites is statistically different between long COVID-19 patients and healthy individuals never infected with SARS-CoV-2. Long COVID-19 patients present lower salivary levels of the metabolites calenduloside G methyl ester, Gly Pro Lys, and creatine than healthy individuals. Lower creatine abundance may be related to reduced physical capacity and fatigue.

## Figures and Tables

**Table 1:** Clinical conditions of long COVID-19 patients during hospitalization.

Variables	
Hospitalization period (days, n = 30)	10.4 ± 6.16
4C score (n = 29)	9 (1–13)
SOFA (n = 21)	2 (0–9)
SAPS III (n = 19)	14.5 (0–46)
APACHE II (n = 20)	8 (1–39)
Mechanical ventilation, %	13
Renal replacement therapy, %	10

4C score: ISARIC 4C mortality score; SOFA: sequential organ failure assessment; SAPS III: simplified acute physiology score version III; APACHE II: acute physiology and chronic health evaluation; n: sample size. Values expressed as mean ± standard deviation or median and percentiles. The color in the table footer is used to separate rows.

**Table 2:** Characteristics of long COVID patients.

Variables	n = 30
Gender F/M (n)	10/20
Age (years)	60.4 ± 14.4
Weight (kg)	89.8 ± 19.8
Height (cm)	166 ± 9.5
BMI (kg/m^2^)	32.2 ± 7.04
Fat-free mass (kg)	30.7 ± 7.30
Fat mass (kg)	34.4 ± 13.7
MIP (cm H_2_O; n = 26; %)	90.1 ± 31.6
MEP (cm H_2_O; n = 26; %)	97.3 ± 31.0
Handgrip (R; %)	35.5 ± 12.4
Handgrip (L; %)	30.7 ± 11.3
SGRQ, total (%)	24.3 ± 14.9
MMRC (score)	0.86 ± 0.97
BDI	9.5 ± 2.62
6mWT (m; n = 24)	418 ± 130

Values expressed as mean ± standard deviation. F: female; M: male; % of predictive: percentage of predictive; R: right; L: left; BMI: body mass index; MIP: maximal inspiratory pressure; MEP: maximal expiratory pressure; SGRQ: Saint George’s Respiratory Questionnaire; MMRC: Modified Medical Research Council scale; BDI: Baseline Dyspnea Index; 6mWT: six-minute walk test. The color in the table footer is used to separate rows.

**Table 3:** Serum biochemical and arterial gases parameters.

Variables	n = 30
White blood cells (×10^3^/mm^3^)	6.68 ± 2.05
D-dimer (ng/mL; n = 15)	503 (172–2221)
PaO_2_ (mmHg)	86.1 ± 9.62
PaCO_2_ (mmHg)	34.8 ± 3.87
SaO_2_ (%)	96.7 ± 1.45

Values expressed as mean ± standard deviation or median and percentiles. D-dimer: A biomarker. PaO_2_: arterial oxygen pressure; PaCO_2_: arterial carbon dioxide pressure; SaO_2_: arterial oxygen saturation. The color in the table footer is used to separate rows.

## Data Availability

The data presented in this study are available on request from the corresponding author. The data are not publicly available due to ethics restrictions.
